# Enhanced P-Type GaN Conductivity by Mg Delta Doped AlGaN/GaN Superlattice Structure

**DOI:** 10.3390/ma14010144

**Published:** 2020-12-31

**Authors:** Ying Zhao, Shengrui Xu, Hongchang Tao, Yachao Zhang, Chunfu Zhang, Lansheng Feng, Ruoshi Peng, Xiaomeng Fan, Jinjuan Du, Jincheng Zhang, Yue Hao

**Affiliations:** 1 Wide Band-Gap Semiconductor Technology Disciplines State Key Laboratory, School of Microelectronics, Xi’dian University, Xi’an 710071, China; yingzi8883@163.com (Y.Z.); hchtao@126.com (H.T.); ychzhang@xidian.edu.cn (Y.Z.); cfzhang@xidian.edu.cn (C.Z.); pengrs9@163.com (R.P.); 18792900253@163.com (X.F.); jinjuan5826@163.com (J.D.); jchzhang@xidian.edu.cn (J.Z.); yhao@xidian.edu.cn (Y.H.); 2 School of Mechanical-Electrical Engineering, Xi’dian University, Xi’an 710071, China; fenglansheng@xidian.edu.cn

**Keywords:** GaN, p-conductivity, superlattice, delta doping, LED

## Abstract

A method of combining the AlGaN/GaN superlattices and Mg delta doping was proposed to achieve a high conductivity p-type GaN layer. The experimental results provided the evidence that the novel doping technique achieves superior p-conductivity. The Hall-effect measurement indicated that the hole concentration was increased by 2.06 times while the sheet resistivity was reduced by 48%. The fabricated green-yellow light-emitting diodes using the achieved high conductivity p-type GaN layer showed an 8- and 10-times enhancement of light output power and external quantum efficiency, respectively. The subsequent numerical calculation was conducted by using an Advanced Physical Model of Semiconductor Device to reveal the mechanism of enhanced device performance. This new doping technique offers an attractive solution to the p-type doping problems in wide-bandgap GaN or AlGaN materials.

## 1. Introduction

III-Nitride and related alloys are attractive materials due to their immense potential in optoelectronic applications including the ultraviolet and visible range [1,2,3,4,5,6,7,8,9]. The well-known Mg is generally used as p-type dopant for GaN based devices. However, Mg has a large acceptor ionization energy E_A_ (~200 meV) at room temperature which is much larger than the donor (Si generally) activation energy E_D_ (~15 meV) in GaN [10]. Therefore, lower acceptor ionization rate becomes an obstacle to obtain high conductivity p-type GaN. P-type GaN was earlier realized by electron irradiation beam and thermal annealing, respectively [11,12], but so far, a high conductivity p-GaN (>10^18^ cm^−3^) epilayer is still difficult to achieve. Heavy Mg doping seems like the most effective way to achieve a high hole concentration of p-type GaN epilayer, but the hole mobility will decrease due to the serious impurity scattering. Furthermore, the increased dopant concentration will lead to a degradation of GaN epilayer crystalline quality. Various methods have been proposed to improve p-conductivity in GaN. Kozodoy et al. utilized the AlGaN/GaN superlattice (SLs) structure only doping in the interface to promote the Mg accepter ionization [13]. However, this discontinuous doping method will lead to the existence of discontinuous high resistivity areas. Simon et al. proposed a polarization-induced hole doping method with a compositionally graded Al*_x_*Ga_1−*x*_N layer [10]. However, the polarization electric field is not strong enough to ionize a large number of Mg dopants. In recent years, there have been many other attempts to increase hole concentration in GaN [14,15,16,17]. In this work, a method of combining the AlGaN/GaN SLs and Mg delta doping is proposed to achieve high conductivity p-type GaN, which is realized by accepter doping in the whole p-type region but increasing the doping concentration at the AlGaN/GaN interface to improve the p-type conductivity. This method not only resolves the problem of existence of high resistance areas of discontinuous doping method, but also utilizes the polarized electric field adequately. Furthermore, a systematical study about the influence of the new p-type technique on the performance of green-yellow light emitting diodes (LED) is performed both numerically and experimentally.

## 2. Materials and Methods 

**Figure 1:**
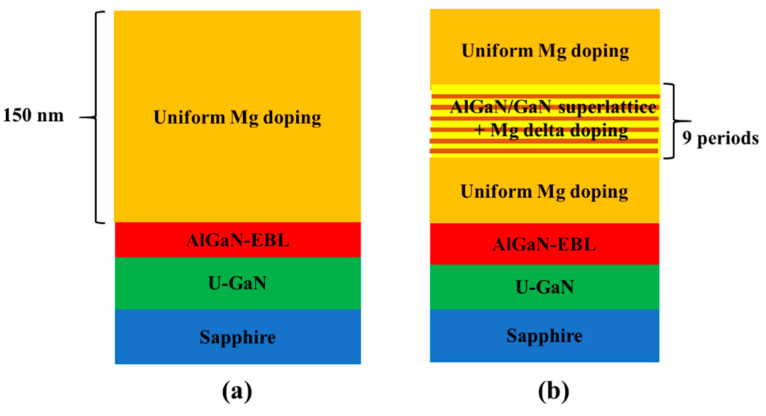
Structure diagrams of p-GaN: (**a**) sample A and (**b**) sample B.

**Figure 2:**
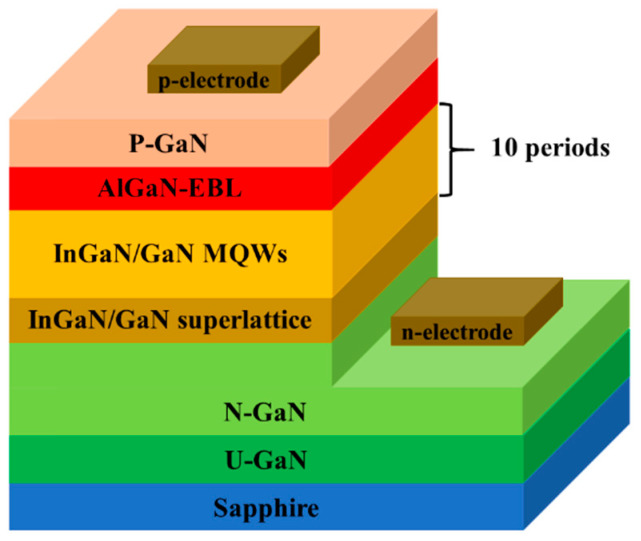
Structure diagram of green-yellow LED.

In order to investigate the influence of novel doping method on the p-conductivity of GaN film, two p-GaN structures were grown by AIXTRON CRUIS I metal-organic chemical vapor deposition (MOCVD) system on the c-plane patterned sapphire substrates (PSS). Figure 1a–b show the two p-GaN sample structures which are named sample A and sample B, respectively. The structures of two p-GaN are identical before Mg doping p-type layer, which contain unintentionally doped GaN layer, Al_0.05_Ga_0.95_N electron blocking layer (EBL), and Mg doped p-type layer. The p-type layer for sample A was uniform Mg doped GaN layer which was used as reference sample. Triethylgallium, trimethylaluminium, and ammonia were used as source compounds. Hydrogen gas was the carrier gas. Bis-cyclopentadienyl-magnesium (CP_2_Mg) was used as a Mg dopant source and the CP_2_Mg flow was kept at 1500 sccm. For sample B, the p-type layer was divided into three parts. The top and bottom parts were uniform Mg doping GaN layer. A 9-periods p-type 4-nm-Al_0.05_Ga_0.95_N/3-nm-GaN structure was adopted in the middle part of sample B. In order to achieve delta doping at the SLs structure in sample B, the flow of CP_2_Mg was constant throughout the entire p-type layer growth, while the growth process had a 6-s pause at each SL interface and the Mg source was still opened. This growth process leads to an intentional increased concentration of Mg dopants at the SLs interfaces and actually implements a Mg delta doping in the middle part of sample B. To further verify the improvement of device performance by novel doping technique, two green-yellow light emitting diodes (LED) are fabricated using the two different p-type GaN layer. Figure 2 shows the structure diagram of the InGaN/GaN multi-quantum wells (MQWs) based LED structure used in this work. An undoped GaN layer is followed by a Si doped GaN layer. The 60-nm-thick In_0.05_Ga_0.95_N/GaN SLs are employed to regulate the stress. The InGaN/GaN MQWs consist of ten 2 nm In_0.45_Ga_0.64_N quantum wells embeds in eleven 15 nm GaN quantum barriers. The In_0.45_Ga_0.64_N/GaN MQWs are terminated by an Al_0.05_Ga_0.95_N EBL. The top of the LED structure is the Mg doped p-type layer. The LED A adopts the uniform Mg doped p-GaN layer while LED B adopts the p-type layer structure of sample B above. After the growth, standard chip processing is carried out and the size of the LED chip is 300 μm × 325 μm. In order to reveal the mechanism of enhanced p-conductivity and device performance, an Advanced Physical Model of Semiconductor Device (APSYS) simulation is adopted based on the structure above. The main parameters set in the simulation are the polarization shielding coefficient of 0.5, the auger composite coefficient of 1 × 10^−43^/(m^6^·s), the band offset ratio of 0.7 and the carrier lifetime of 10 ns.

## 3. Results and Discussions

**Figure 3:**
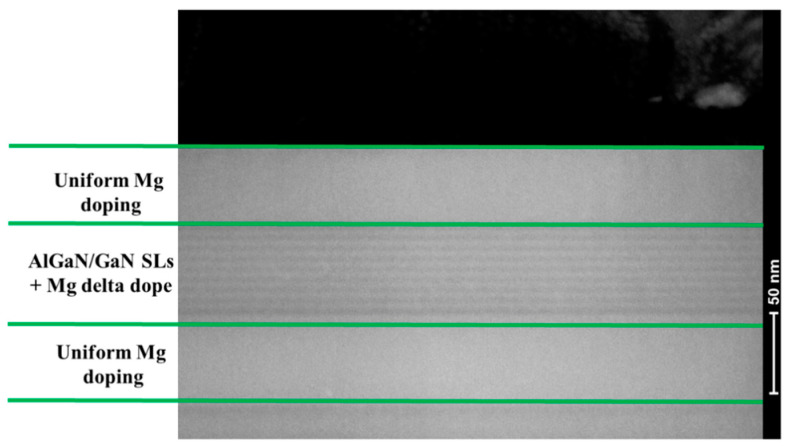
Cross-sectional TEM image of the p-type layer of sample B.

Figure 3 shows the cross-sectional transmission electron microscope (TEM) image of sample B, the p-GaN layer and the structure of AlGaN/GaN SLs can be clearly observed. The TEM image was acquired using a Tecnai G2 F20 S-Twin (FEI, Hillsboro, OR, USA) operating at 200 kV. 

To characterize the different conductivities of the two p-GaN samples, the Hall-effect measurement was performed at room temperature using a Lake Shore 8400 Series at a magnetic field of 0.5 T. The measured hole concentrations, hole mobilities, and sheet resistivities are shown in Table 1. From the Hall-effect measurements, the hole concentration of sample B is 3.1 × 10^18^ cm^−3^, which is 2.06 times higher than that of sample A, and it is an order of magnitude higher than previous reported hole concentration of p-GaN grown by MOCVD [18,19,20]. At the same time, as shown in Table 1, the increased impurities and interface scattering at interface of AlGaN/GaN SLs in sample B also lead to a decrease of hole mobility, but the sheet resistivity still decreases significantly due to the large hole concentration. 

**Figure 4:**
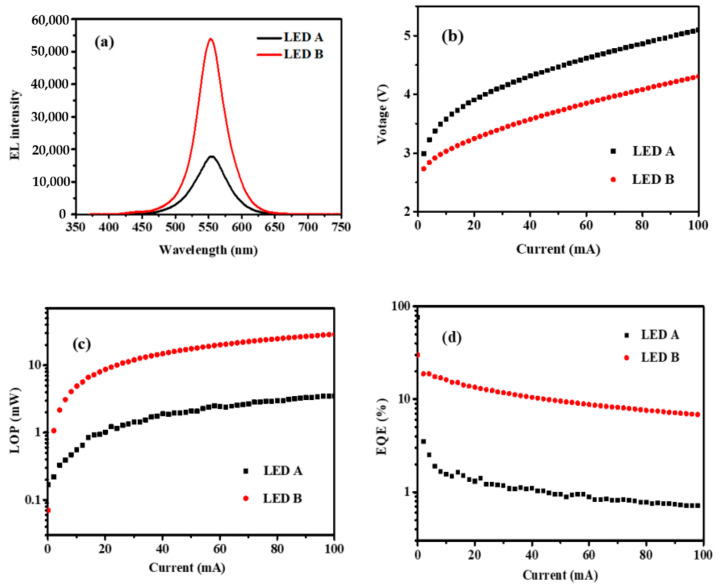
(**a**) Electroluminescence (EL) spectra, (**b**) Voltage, (**c**) light output power (LOP), and (**d**) external quantum efficiency (EQE) as a function of the injection direct current of the green-yellow LED, respectively.

In order to verify the effectiveness of p-conductivity on the performance of LED, Figure 4a shows electroluminescence (EL) spectra of LED A and LED B at the injection current of 20 mA. As illustrated in Figure 4a, the peak emission wavelengths of both samples are around 559 nm and the EL intensity of LED B is significantly enhanced compared with that of LED A. Figure 4b,d show the voltage, light output power (LOP) and external quantum efficiency (EQE) as functions of the injection current. It can be clearly observed that the voltage of LED B is significantly lower than that of LED A at the same injection current. The LOP and EQE of LED B have about an order of magnitude improvement than that of the LED A. At an injection current of 20 mA, the peak wavelengths are 559.3 and 559.6 nm for LED A and LED B, respectively. The LOP of LED A is 1.03 mW and the EQE is 1.32% while LED B has a higher LOP and EQE which are 8.7 mW and 13.4%. Obviously, the improved p-conductivity significantly enhanced the performance of GaN based LED. In previous work, the optimization of the growth conditions and MQWs structures were proposed to achieve high performance GaN based LED. Lai et al. demonstrated a high-efficiency yellow-green LED with the peak wavelength of 560.7 nm at 20 mA. The reported EQE was 2.1%, which is significantly lower than LED B [21]. Saito et al. reported a LED on *c*-face sapphire substrate with wavelength of 559 nm, the LOP was 11 mW at an injection current of 20 mA [22]. However, the voltage of the report LED was 5.71 V when the injection current is 20 mA, which is much higher than the LED B in this work (3.9 V). Du et al. fabricated a LED chip on *c*-plane sapphire substrate with a size of 300 × 300 μm^2^. But the dominant wavelength and output power at 20 mA were 556.3 nm and 0.24 mW, respectively [23]. Compared with the previous reports, the performance of LED B has obvious advantages, which demonstrates that the novel technique of p-type doping in this work is very promising for the achievement of high-efficiency GaN based LED.

**Figure 5:**
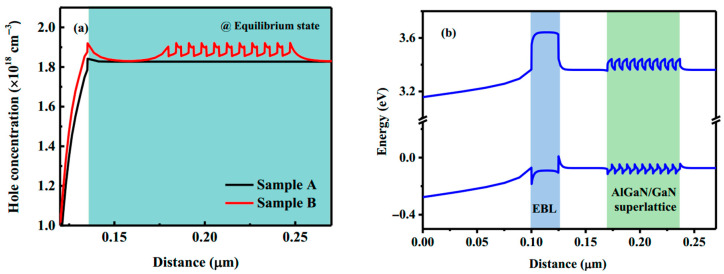
(**a**) Simulated hole concentration of both sample A and sample B, and (**b**) Simulated energy-band diagram of sample B.

In order to clarify the mechanism of novel doping methods enhanced the p-conductivity of GaN film, the calculated hole concentrations distributions and energy band diagrams of sample B are given in Figure 5, which are performed by the commercial simulation software APSYS. Figure 5a shows the simulated hole concentration of both sample A and sample B, which indicated that the hole concentration is significantly increased in the middle layer of sample B by the novel p-doping structure. To illustrate the mechanism of hole concentration enhancement, the simulated energy band of sample B is shown in Figure 5b, and the up and down blue lines correspond to the conduction and valence band edges, respectively. The periodic AlGaN/GaN SLs structure generated a periodic oscillation of the energy-band edge can be observed. The sharp discontinuity of the polarization field at abrupt AlGaN/GaN heterojunction interface leads to the formation of a bound sheet charge at the heterointerface and generated a strong electric field. Because the Mg acceptors are favorable to ionize at the band edge trending down, the intentionally increased Mg dopants at SL interface will effectively utilize the polarization field to increases the ionization rate of Mg dopant and enhance the hole concentration [24]. 

**Figure 6:**
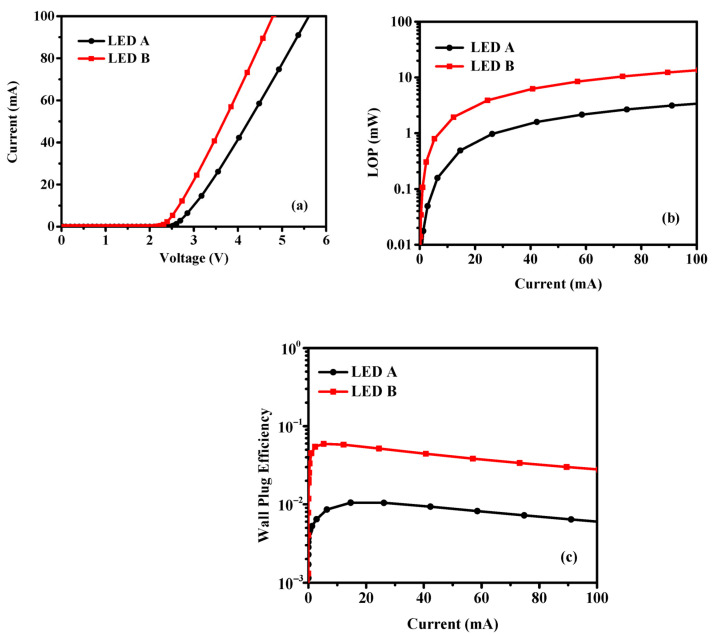
Advanced Physical Model of Semiconductor Device (APSYS) simulated results of LED A and LED B: (**a**) Voltage, (**b**) LOP and (**c**) WPE as a function of the injection direct current.

In addition, for take a deep insight into the improved p-conductivity on the performance of LED, the APSYS simulated results of LED performance is presented in Figure 6. Figure 6a–c show the simulated I-V curves, light output power (LOP), and wall plug efficiency (WPE) varied with injection current. It is clearly that the LED B exhibit lower voltage, higher LOP and WPE under the same injection current compared with LED A. The simulated results implied that the LED performance is enhanced due to the adoption of the novel p-doping structure, which is consistent with the experimental results.

## 4. Conclusions

In summary, we proposed a method of combining the AlGaN/GaN SLs and Mg delta doping to achieve a high conductivity p-type GaN. Hall-effect measurement showed the hole concentration of sample B was increased by 2.06 times while the sheet resistivity was reduced by 48% compared to sample A. In order to verify the enhancement of the GaN based photoelectric device performance by improved p-conductivity, a green-yellow LED was fabricated using the optimized high conductivity p-GaN layer and the EQE was increased by 10 times. The APSYS simulated results illustrated the enhancement mechanism of p-conductivity and LED performance. The novel technique of p-type doping in this work are very promising for the achievement of high-efficiency GaN based LEDs and electronic devices.

## Figures and Tables

**Table 1:** Results of the Hall measurement of the samples at room temperature.

p-GaN	Hole Concentration (×10^18^ cm^−3^)	Mobility (cm^2^/V·s)	Sheet Resistivity (Ω/□)
Sample A	1.5	10.0	34,407
Sample B	3.1	7.5	17,951
